# Novel Osteogenic Behaviors around Hydrophilic and Radical-Free 4-META/MMA-TBB: Implications of an Osseointegrating Bone Cement

**DOI:** 10.3390/ijms21072405

**Published:** 2020-03-31

**Authors:** Yoshihiko Sugita, Takahisa Okubo, Makiko Saita, Manabu Ishijima, Yasuyoshi Torii, Miyuki Tanaka, Chika Iwasaki, Takeo Sekiya, Masako Tabuchi, Naser Mohammadzadeh Rezaei, Takashi Taniyama, Nobuaki Sato, Juri Saruta, Masakazu Hasegawa, Makoto Hirota, Wonhee Park, Masaichi Chang-Il Lee, Hatsuhiko Maeda, Takahiro Ogawa

**Affiliations:** 1 Weintraub Center for Reconstructive Biotechnology, Division of Advanced Prosthodontics, UCLA School of Dentistry, Los Angeles, CA 90095-1668, USA; yosshii@dpc.agu.ac.jp (Y.S.); okubotakahisa@gmail.com (T.O.); saita@kdu.ac.jp (M.S.); manab612@gmail.com (M.I.); ya3w4tt@gmail.com (Y.T.); minkey646@gmail.com (M.T.); chikaiwsk@gmail.com (C.I.); yuko_ta_01@yahoo.co.jp (T.S.); machako@dpc.aichi-gakuin.ac.jp (M.T.); naser.m.rezaei@gmail.com (N.M.R.); taniyama.orth@tmd.ac.jp (T.T.); nobuakisato19@gmail.com (N.S.); saruta@kdu.ac.jp (J.S.); masa.hasegawa0202@gmail.com (M.H.); mhirota@yokohama-cu.ac.jp (M.H.); drwon69@gmail.com (W.P.); 2 Department of Oral Pathology, School of Dentistry, Aichi Gakuin University, 1-100 Kusumoto-cho, Chikusa-ku, Nagoya, Aichi 464-8650, Japan; hatsu@dpc.aichi-gakuin.ac.jp; 3 Department of Oral Interdisciplinary Medicine (Prosthodontics & Oral Implantology), Graduate School of Dentistry, Kanagawa Dental University, 82 Inaoka, Yokosuka, Kanagawa 238-8580, Japan; 4 Department of Orthopedic Surgery, Yokohama City Minato Red Cross Hospital, 3-12-1 Shinyamashita, Yokohama, Kanagawa 231-8682, Japan; 5 Department of Oral Science, Graduate School of Dentistry, Kanagawa Dental University, 82 Inaoka, Yokosuka, Kanagawa 238-8580, Japan; 6 Yokosuka-Shonan Disaster Health Emergency Research Center and ESR Laboratories, Graduate School of Dentistry, Kanagawa Dental University, 82 Inaoka, Yokosuka, Kanagawa 238-8580, Japan; lee@kdu.ac.jp

**Keywords:** arthroplasty, total hip replacement, free radical, PMMA, cytotoxicity, implants

## Abstract

Poly(methyl methacrylate) (PMMA)-based bone cement, which is widely used to affix orthopedic metallic implants, is considered bio-tolerant but lacks osteoconductivity and is cytotoxic. Implant loosening and toxic complications are significant and recognized problems. Here we devised two strategies to improve PMMA-based bone cement: (1) adding 4-methacryloyloxylethyl trimellitate anhydride (4-META) to MMA monomer to render it hydrophilic; and (2) using tri-n-butyl borane (TBB) as a polymerization initiator instead of benzoyl peroxide (BPO) to reduce free radical production. Rat bone marrow-derived osteoblasts were cultured on PMMA-BPO, common bone cement ingredients, and 4-META/MMA-TBB, newly formulated ingredients. After 24 h of incubation, more cells survived on 4-META/MMA-TBB than on PMMA-BPO. The mineralized area was 20-times greater on 4-META/MMA-TBB than PMMA-BPO at the later culture stage and was accompanied by upregulated osteogenic gene expression. The strength of bone-to-cement integration in rat femurs was 4- and 7-times greater for 4-META/MMA-TBB than PMMA-BPO during early- and late-stage healing, respectively. MicroCT and histomorphometric analyses revealed contact osteogenesis exclusively around 4-META/MMA-TBB, with minimal soft tissue interposition. Hydrophilicity of 4-META/MMA-TBB was sustained for 24 h, particularly under wet conditions, whereas PMMA-BPO was hydrophobic immediately after mixing and was unaffected by time or condition. Electron spin resonance (ESR) spectroscopy revealed that the free radical production for 4-META/MMA-TBB was 1/10 to 1/20 that of PMMA-BPO within 24 h, and the substantial difference persisted for at least 10 days. The compromised ability of PMMA-BPO in recruiting cells was substantially alleviated by adding free radical-scavenging amino-acid N-acetyl cysteine (NAC) into the material, whereas adding NAC did not affect the ability of 4-META/MMA-TBB. These results suggest that 4-META/MMA-TBB shows significantly reduced cytotoxicity compared to PMMA-BPO and induces osteoconductivity due to uniquely created hydrophilic and radical-free interface. Further pre-clinical and clinical validations are warranted.

## 1. Introduction

Bone fractures and degenerative joint changes related to osteoporosis and arthritis are common problems in elderly patients and their incidence is increasing as the population ages. For example, there are currently 8.8 million osteoporotic fractures each year worldwide [1]. When bone and joint fractures fail to heal naturally, metallic implants are used for immobilization, restoration, and reconstruction, and bone cement is used to stabilize the implants in a significant proportion of these procedures. However, implant loosening remains the most important complication, resulting in a high incidence of revision surgeries and considerable patient morbidity [2,3,4,5,6,7].

Bone cement, which is used to fill the gap between the implant and bone, is usually a mixed solid and liquid resin: the solid part is pre-polymerized polymethyl methacrylate (PMMA) and the liquid part is methyl methacrylate (MMA). Implant stability is achieved by the mechanical interlocking of bone and bone cement rather than biological adhesion or integration. PMMA-based bone cement does not induce new bone formation due to a lack of osteoconductivity; instead, fibrous soft tissue forms around bone cement as result of an inflammatory reaction [8,9,10,11]. Moreover, bone cement can induce multiple adverse tissue reactions including impaired bone remodeling, necrosis, fibrosis, and histiocytosis, which may directly cause implant loosening and failure [12,13]. At the cellular level, bone cement inhibits osteoblast proliferation and function [14,15,16] and induces cellular apoptosis and necrosis [17]. A dire adverse systemic complication known as bone cement implantation syndrome (BCIS) is a critical concern. BCIS is characterized by hypotension, hypoxemia, cardiac arrhythmias, cardiac arrest, or their combination and can cause immediate death (0.6%–1% in some recipient groups) [18,19].

These unfavorable effects of PMMA-based bone cements may arise from oxidative stress at the cellular and tissue levels created by the necessary production of free radicals triggered by the peroxide initiator [20,21,22], often benzoyl peroxide (BPO) for current PMMA-based bone cement products. Indeed, osteoblasts exposed to BPO-containing PMMA resin show a high percentage of cell death and severely compromised proliferation and differentiation [23,24]. However, neutralizing the free radicals with antioxidants restores impaired osteoblastic function, proving that controlling polymerization free radical production may hold a key to developing more biocompatible bone cement [23,24,25].

We here devised two strategies to improve PMMA-based bone cement: (1) adding hydrophilicity to bone cement to increase cellular affinity; and (2) using a different type of polymerization initiator to minimize free radical production. With respect to hydrophilicity, there is evidence that hydrophilic titanium surfaces attract osteoblasts and promote subsequent osteogenesis [26,27,28,29,30,31]. To make PMMA more hydrophilic, here we added 4-methacryloyloxylethyl trimellitate anhydride (4-META) to the MMA monomer, since 1%–5% 4-META added to conventional acrylic resin is known to form hydrophilic methacrylate [32,33,34]. With respect to minimizing free radical production, we tested tri-n-butyl borane (TBB) as a polymerization initiator. TBB better promotes polymerization than BPO, leaving less residual monomer [35] while suppressing the production of free radicals [36]. In addition, TBB-initiated polymerization generates less heat than BPO [37]. Further, unlike BPO, TBB is moisture resistant, and the addition of TBB may help promote polymerization under wet conditions [34,38], a valuable property for bone cement used in bone marrow cavities. Given these strategies and supporting rationale, the objective of this study was to compare the biological capability and osteoconductivity of PMMA-BPO, common bone cement ingredients, and 4-META/MMA-TBB, newly formulated ingredients. To gain an understanding of the underlying mechanisms, the physicochemical properties of these materials, i.e., time-dependent changes in hydrophobic/hydrophilic properties and the production of polymerization radicals were also studied.

## 2. Results

### 2.1. Material Characterization

We confirmed the progress and completion of polymerization by conducting thermodynamic, chemical, morphologic, and mechanical characterization. The peak temperature was clearly identified for both PMMA-BPO and 4-META/MMA-TBB materials. The temperature of PMMA-BPO peaked at 45.9 °C 4 min and 40 s after mixing, while the temperature of 4-META/MMA-TBB peaked at 38.5 °C 7 min after mixing. The degree of polymerization measured by FT-IR was 70.8% and 76.1% for PMMA-BPO and 4-META/MMA-TBB materials, respectively, 1 h after mixing and increased to 87.8% and 85.2%, respectively, at 24 h.

**Figure 1:**
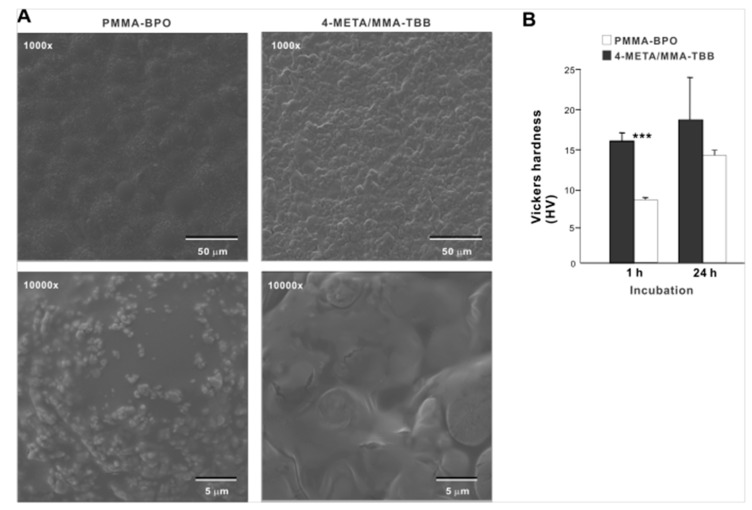
Morphologic and mechanical characterization of polymer materials used in this study. (**A**) SEM images of PMMA-BPO and 4-META/MMA-TBB materials 24 h after mixing polymer and monomer. (**B**) Vickers hardness of the two materials 1 and 24 h after mixing. *** *p* < 0.001, statistically significant difference between the two materials. PMMA-BPO, poly(methyl methacrylate)–benzoyl peroxide; 4-META, 4-methacryloyloxylethyl trimellitate anhydride; MMA-TBB, methyl methacrylate–tri-n-butyl borane.

Low-magnification SEM images of PMMA-BPO 24 h after mixing showed smooth texture with hemi-spherical structures ranging from 30 to 40 µm in diameter, suggestive of polymer particles embedded in the polymerized material (Figure 1A). The high magnification images showed sub-micron scale structures in undefined form scattered all over the PMMA-BPO surface. The 4-META/MMA-TBB showed even and uniform morphology with finer projecting features with their size of approximately 10 µm. There was no polymer particle exposed. The high-magnification images of 4-META/MMA-TBB did not show the sub-micron scale deposits. These results from thermodynamics, FT-IR-assisted molecular, and morphologic characterization collectively and consistently confirmed the successful polymerization of the two materials. The Vickers test showed that the surface hardness of 4-META/MMA-TBB was significantly lower than PMMA-BPO at 1 h, whereas the difference was insignificant at 24 h (Figure 1B).

### 2.2. Improved Attachment, Viability, and Initial Behavior of Osteoblasts on 4-META/MMA-TBB

**Figure 2:**
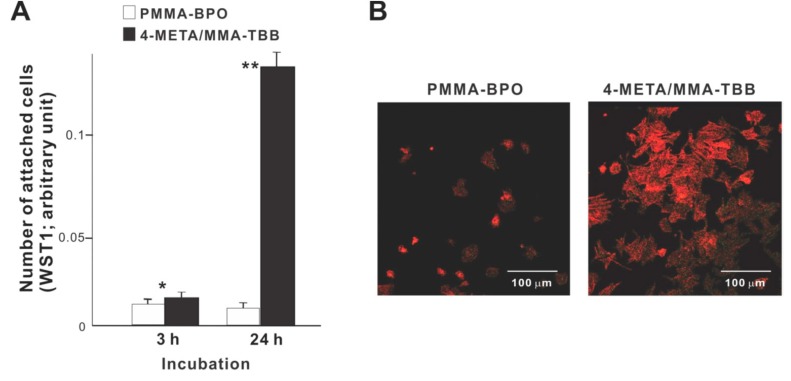
Osteoblast attachment to two different resinous materials during the initial stage of culture. (**A**) The number of cells attached to material surfaces at 3 h and 24 h post-seeding evaluated by the WST-1 assay. * *p* < 0.05, ** *p* < 0.01, statistically significant difference between the two materials. (**B**) Confocal microscopic images of osteoblasts 24 h after seeding on two different materials.

We commenced biological characterization of the two different resins by examining the initial responses of osteoblasts seeded on these materials. Significantly more osteoblasts attached to 4-META/MMA-TBB cement than to PMMA-BPO cement both at 3 and 24 h of culture (Figure 2A). In particular, approximately 15-times more osteoblasts attached to 4-META/MMA-TBB at 24 h. Low-magnification confocal microscopic images at 24 h showed a considerably greater number of cells attached to 4-META/MMA-TBB than to PMMA-BPO (Figure 2B), supporting the result of the colorimetric assay.

**Figure 3:**
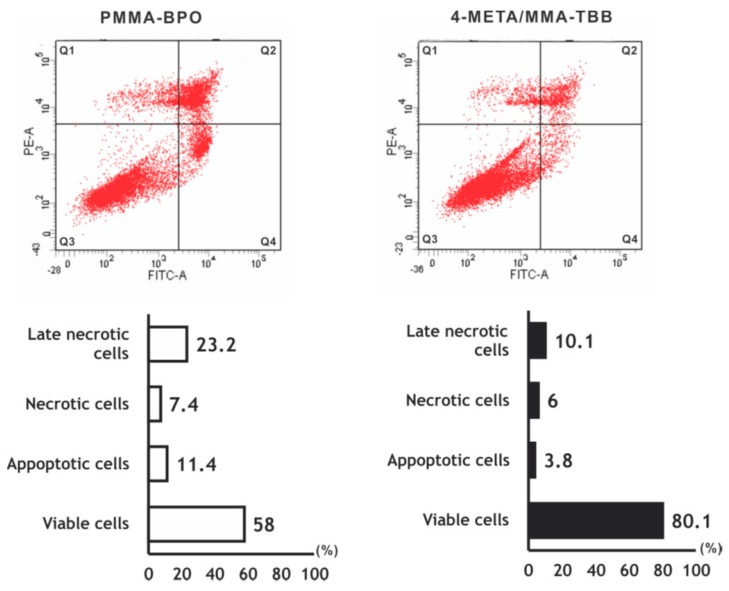
Flow cytometry-based viability/death analysis of osteoblasts seeded on two different materials. Cells 24 h after seeding were analyzed. Flow cytometric images (top) and percentages of viable cells (Q3 in top images), apoptotic cells (Q4), necrotic cells (Q1), and late necrotic cells (Q2).

The number of viable osteoblasts on 4-META/MMA-TBB after 24 h was higher than that on PMMA-BPO (Figure 3), with 80.1% on 4-META/MMA-TBB and 58.0% on PMMA-BPO. The percentage of apoptotic cells and late necrotic cells was lower on 4-META/MMA-TBB cement.

**Figure 4:**
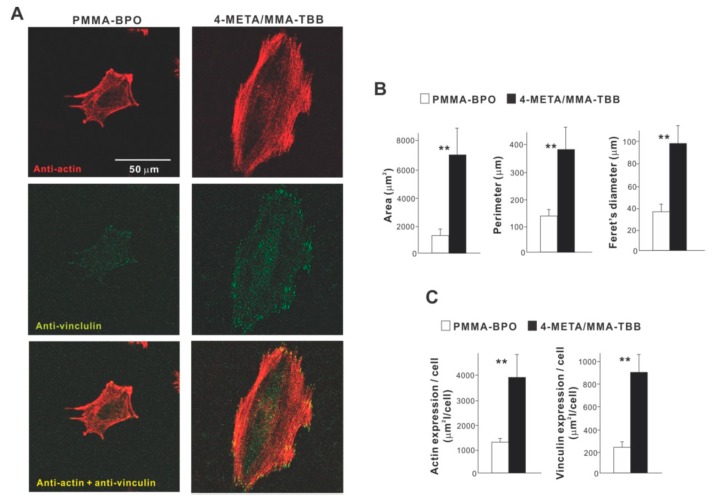
Attachment and spreading behavior of osteoblasts on two different materials. (**A**) Confocal microscopic images of osteoblasts with immunochemical staining for cytoskeletal actin and the adhesion protein vinculin are shown. Cytomorphometric (**B**) and densitometric (**C**) parameters measured from the images are presented. ** *p* < 0.01, statistically significant difference between the two materials.

High magnification confocal microscopy at 24 h showed that osteoblasts on 4-META/MMA-TBB were generally spread larger and had more intensive localization of cytoskeletal actin along the cellular outline, suggestive of advanced lamellipodia-like cytoplasmic projection development (Figure 4A). Additionally, the expression of a focal adhesion protein, vinculin, appeared denser and more extensive in osteoblasts seeded on 4-META/MMA-TBB cement. Cytomorphometric (Figure 4B) and densitometric (Figure 4C) evaluations confirmed these qualitative observations.

### 2.3. Improved Proliferation and Functional Phenotype of Osteoblasts on 4-META/MMA-TBB

**Figure 5:**
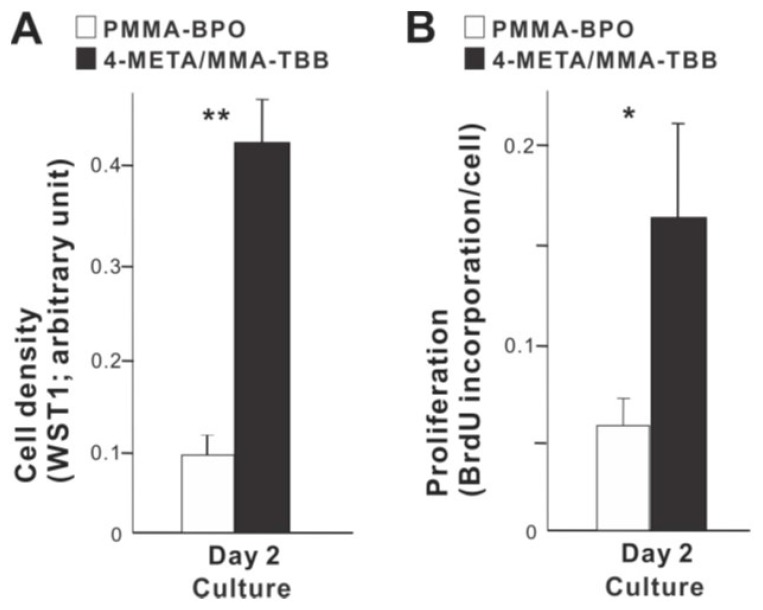
Proliferative activity of osteoblasts on two different materials. (**A**) Cell density evaluated on day 2 using the WST-1 assay. (**B**) The rate of proliferation evaluated on day 2 using the BrdU incorporation assay. * *p* < 0.05, ** *p* < 0.01, statistically significant difference between the two materials.

We next examined how osteoblast proliferation and function are affected by the two different materials. The number of propagated osteoblasts on day 2 of culture was significantly greater on 4-META/MMA-TBB than on PMMA-BPO (Figure 5A). Similarly, the proliferative activity as measured by BrdU incorporation into DNA was higher on 4-META/MMA-TBB (Figure 5B). 

**Figure 6:**
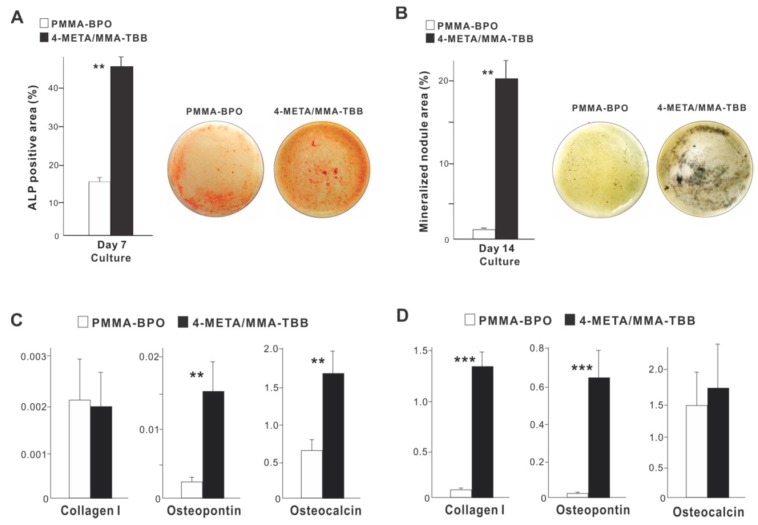
Functional differentiation and phenotypes of osteoblasts cultured on two different materials. (**A**) Images of cultures after alkaline phosphatase (ALP) staining on day 7 and ALP-positive area (%) measured using those images. (**B**) Mineralization capability of osteoblast cultures evaluated on day 14. Photos with von Kossa stain and von Kossa-positive area are shown. The expression of osteogenic genes evaluated by real-time quantitative PCR on days 3 (**C**) and 10 (**D**) of culture. ** *p* < 0.01, *** *p* < 0.001, statistically significant difference between the two materials.

With respect to functional phenotype, ALP activity measured on day 7 was significantly greater on 4-META/MMA-TBB than on PMMA-BPO (Figure 6A). Furthermore, the mineralization activity of osteoblasts was also remarkably increased on 4-META/MMA-TBB on day 14 (Figure 6B).

The expression of osteogenic genes was evaluated on days 3 and 10. Osteopontin and osteocalcin expression was upregulated on 4-META/MMA-TBB on day 3 compared to PMMA-BPO, while the expression of type 1 collagen was similar between the two materials (Figure 6C). On day 10, the expression of type 1 collagen and osteopontin was significantly upregulated on 4-META/MMA-TBB cement (Figure 6D).

### 2.4. Enhanced In Vivo Anchorage and Osteogenic Activity around 4-META/MMA-TBB

**Figure 7:**
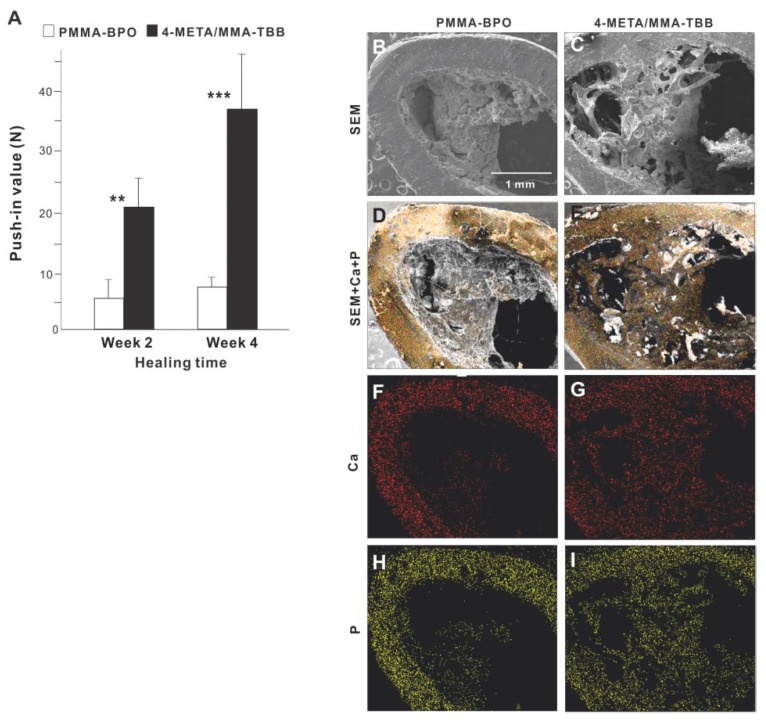
(**A**) The strength of bone-cement integration for two different materials. The strength of integration evaluated by the biomechanical push-in test in the rat femur model at weeks 2 and 4 of healing. ** *p* < 0.01, *** *p* < 0.001, statistically significant difference between the two materials. Tissue morphology and chemistry evaluated after push-in testing (**B**–**I**). Implants with cement materials were retrieved after the biomechanical push-in test and analyzed by SEM (B,C) and EDX (D–I). EDX was performed to map Ca and P elements.

The strength of bone-cement integration as evaluated by the biomechanical push-in test at week 2 of healing was 4-times greater for 4-META/MMA-TBB than for PMMA-BPO (Figure 7A). The difference was even greater (5-times) at week 4. The push-in value significantly increased between 2 and 4 weeks for 4-META/MMA-TBB but not for PMMA-BPO.

SEM images of the implant-cement complex after push-in testing showed that biological structures were more extensively deposited around 4-META/MMA-TBB implants/cement (Figure 7B,C), including an organized trabecular structure originating and extending from the periosteum and bone marrow cavity (Figure 7C). In contrast, the morphology of the PMMA-BPO implant-tissue complex was indistinct and nodular. EDX elemental mapping detected Ca and P across nearly the entire area of the 4-META/MMA-TBB implant complex but only in a limited area of the PMMA-BPO implant complex (Figure 7D–I). The intensity of Ca and P around the 4-META/MMA-TBB implant complex was equivalent to that detectable in cortical bone (Figure 7F–I).

**Figure 8:**
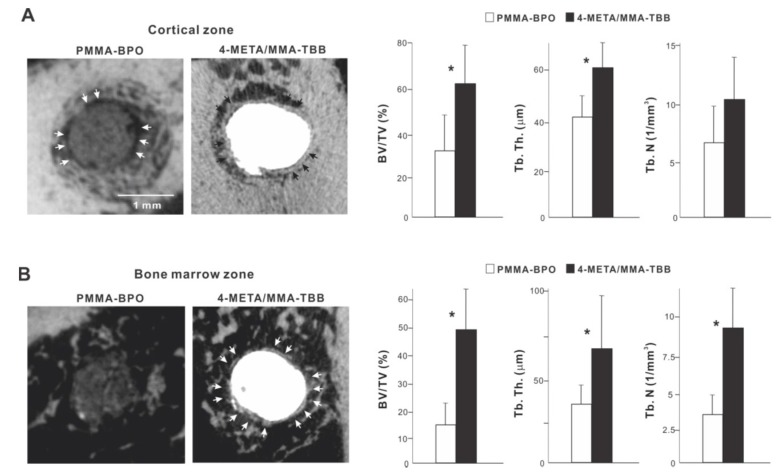
Peri-cement osteogenic behavior around two different materials evaluated by microCT at week 2 of healing. Representative cross-sectional CT images along the long axis of the implant: (**A**) a slice 0.3 mm from the implant top, and (**B**) the bone marrow cavity, a slice 0.3 mm from the implant apex. The quantitative evaluation of bone volume/tissue volume (BV/TV), trabecular thickness (Tb. Th), and trabeculae number (Th. N) are shown for each zone. * *p* < 0.05, statistically significant difference between the two materials.

We continued bone morphometric analysis of the implant-cement complex using microCT. In the cortical zone at week 2 of healing, there was extensive and intimate bone formation along 4-META/MMA-TBB cement (black arrowheads in Figure 8A), with the majority of the newly formed bone juxtaposing the cement. A large part of the gap between the innate cortical bone and implant-cement complex was filled with new bone. In contrast, bone formation seemed to originate from the surrounding cortical bone around PMMA-BPO cement, with little bone formation in the proximity of the PMMA-BPO cement, leaving the gap unfilled (white arrowheads). There was nearly no new bone tissue in direct contact with the PMMA-BPO cement. Quantitative analysis showed that BV/TV and trabecular thickness were significantly greater for the 4-META/MMA-TBB implant/cement complex than for the PMMA-BPO implant/cement complex. 

In the bone marrow zone, bone formation around PMMA-BPO implants/cement appeared limited, scattered, and distant from the cement surface (Figure 8B). In contrast, bone formation around 4-META/MMA-TBB implants/cement was robust, contiguous, and juxtaposed with the cement surface (white arrowheads). BT/TV, trabecular thickness, and trabeculae number were significantly higher around 4-META/MMA-TBB implant/cement than PMMA-BPO implant/cement (Figure 8B).

**Figure 9:**
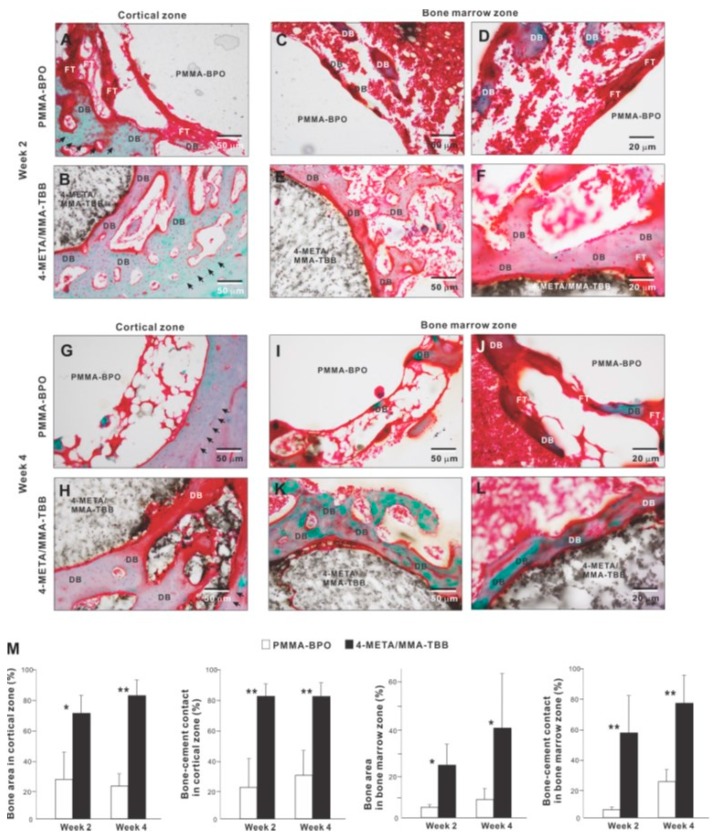
Peri-cement osteogenic morphology and morphometry around two different materials evaluated histologically at weeks 2 and 4 of healing. Representative histological images from week 2 (**A**–**F**) and 4 (**G**–**L**) are shown together with quantitative assessment of bone area and bone-cement contact (**M**). The qualitative and quantitative assessments were performed in both cortical and bone marrow zones. * *p* < 0.05, ** *p* < 0.01, statistically significant difference between the two materials. DB: de novo bone; FT: fibrous tissue.

We further examined peri-cement osteogenic behavior by histomorphometry. Histological images in the cortical zone at week 2 of healing showed that there was a pattern of de novo bone formation around PMMA-BPO implant/cement. The bone formation appeared to originate from the existing cortical bone (black arrowheads in panel A); there was thick fibrous soft tissue or a tissue void between the de novo bone and cement, with only a very limited area of bone in direct contact with the cement (Figure 9A). In contrast, there was extensive and contiguous de novo bone formed around 4-META/MMA-TBB implant/cement near the cement surface as well as distant from the cement (Figure 9B). Bone formation originating both at the existing cortical bone (black arrowheads) and the cement interface were well merged, filling a large part of the surgical gap. There was little fibrous tissue between 4-META/MMA-TBB and bone.

In the bone marrow zone, bone formation around PMMA-BPO was limited and discrete as shown in representative images (Figure 9C,D). A large part of the bone was interposed with thick fibrous tissue (Figure 9D). In contrast, bone formed extensively and contiguously around 4-META/MMA-TBB with little fibrous tissue interposition (Figure 9E,F).

At week 4 of healing, bone formation appeared to extend from the innate cortical bone (black arrowheads) in the PMMA-BPO group. However, the area adjacent to the cement lacked bone, instead showing evidence of a tissue void, fibrous tissue, or blood cells (Figure 9G). Around the 4-META/MMA-TBB cement, new bone juxtaposed the cement surface. A large area of the cement surface was intimately covered by contiguous bone formation with little fibrous tissue interposition. In the bone marrow cavity, the amount of bone formation around the PMMA-BPO implant/cement remained limited and appeared fragmented and scattered (Figure 9I,J). De novo bone tissue was rarely in contact with the cement; instead, the bone fragment was predominantly accompanied by fibrous tissue (Figure 9I,J). In the 4-META/MMA-TBB group, de novo bone was located primarily adjacent to the cement (Figure 9K,L), and a large part of the cement surface was covered by bone. A large part of the bone formed distant from the cement surface at week 2 was remodeled and no longer present, indicating that peri-cement osteogenesis was in its maturation stage (Figure 9K,L).

Histomorphometric analyses demonstrated that bone volume was remarkably greater in the 4-META/MMA-TBB group than the PMMA-BPO group both in the cortical and bone marrow zones at weeks 2 and 4 (Figure 9M). Likewise, the percentage of bone-cement contact in the cortical and bone marrow zones was much greater in the 4-META/MMA-TBB group, supporting the microscopic findings.

### 2.5. Sustained Hydrophilicity and Suppression of Polymerization Radical Production on 4-META/MMA-TBB

**Figure 10:**
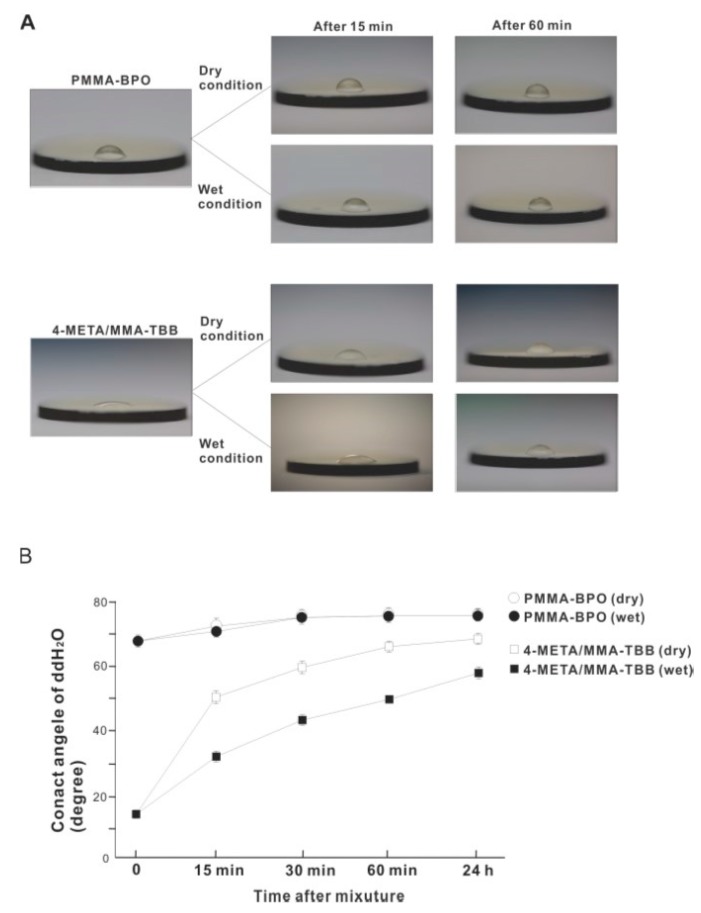
Hydrophilic/hydrophobic property of two different material surfaces and their time-dependent changes. (**A**) Bird’s eye-view images of 10 μL ddH_2_O placed on the materials at different time points after mixing. (**B**) A line graph of the contact angle of ddH_2_O plotted against time after mixing.

The contact angle of 10 μL of ddH_2_O placed on the surface of 4-META/MMA-TBB immediately after mixing was 14.5°, which is considered hydrophilic (contact angle <60°) (Figure 10A,B). The 4-META/MMA-TBB surface remained hydrophilic until 15 min after mixing under dry conditions. In contrast, the contact angle of ddH_2_O on PMMA-BPO was greater than 65° immediately after mixing and was considered hydrophobic. The contact angle increased to greater than 70° 15 min after mixing under dry conditions and stayed hydrophobic up to 24 h after mixing. The contact angle on the 4-META/MMA-TBB surface remained below 60° for up to 1 h under wet conditions and remained hydrophilic. The sustained hydrophilicity under wet conditions was not observed for PMMA-BPO, which remained hydrophobic regardless of condition and time observed.

**Figure 11:**
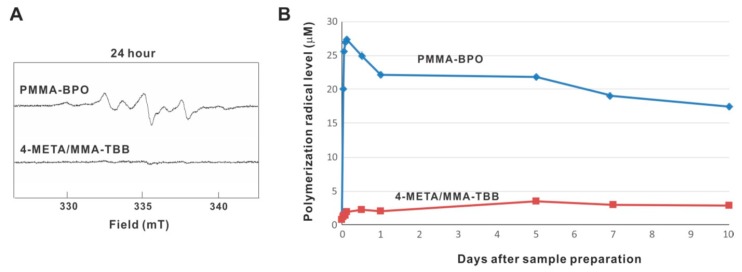
Free radical generation in polymerizing two different materials evaluated by electron spin resonance spectroscopy (ESR). (**A**) ESR spectrums recorded 24 h after mixing materials. (**B**) The time course of free radical production up to 10 days for each material.

ESR analysis revealed that free radical production rapidly increased immediately after mixing within 1 h inside PMMA-BPO, and that the production remained high throughout and up to 10 days, even though production decreased slightly over time (Figure 11A,B). On 10 days, free radical production remained approximately 80% of its peak. Free radical production within 4-META/MMA-TBB also increased within 1 h but remained very low throughout and up to 10 days, in marked contrast to PMMA-BPO. The free radical level for 4-META/MMA-TBB was approximately 1/25th that seen for PMMA-BPO 1 h post-mixing, 1/10th that of PMMA-BPO 1-day post-mixing, and 1/6th that of PMMA-BPO 10-days post-mixing.

### 2.6. Restored Function of PMMA-BPO by Incorporating Anti-Oxidant Molecules

**Figure 12:**
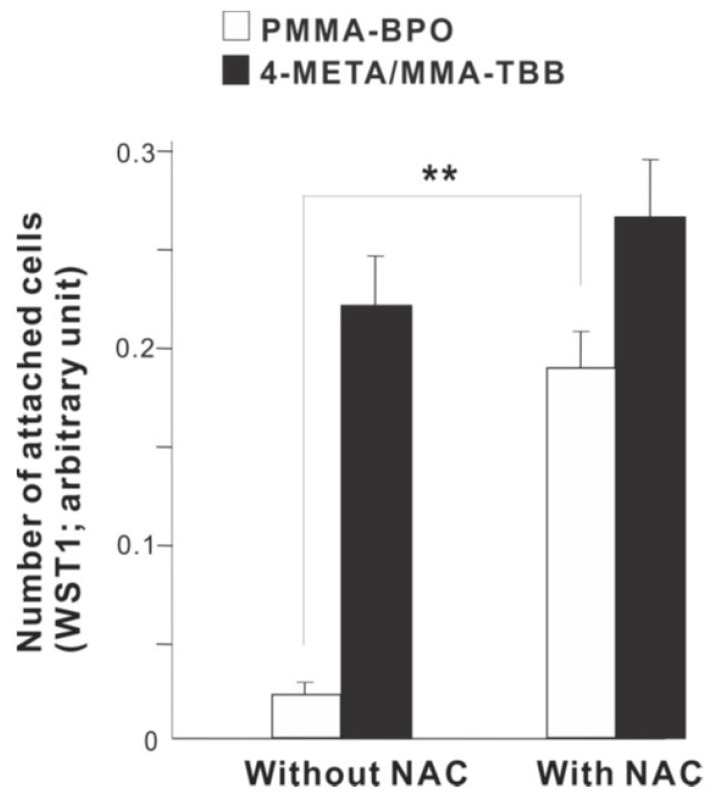
A rescue attempt of two different materials by incorporating anti-oxidant amino acid derivative, N-acetyl cysteine (NAC), into the materials examined by the ability of the materials to facilitate cell attachment. The number of osteoblasts attached 24 h after seeding evaluated by WST-1 assay is shown. ** *p* < 0.01, statistically significant difference.

To examine the role of free radical production, we determined the effect of incorporating anti-oxidant amino acid, NAC into the materials. The number of osteoblasts attached 24 h after seeding was considerably increased on PMMA-BPO with NAC compare to PMMA-BPO without NAC (Figure 12). In contrast, adding NAC did not significantly influence the degree of cell attachment on 4-META/MMA-TBB.

## 3. Discussion

These results comprehensively and consistently support the hypothesis that adding 4-META to MMA and the use of TBB as an initiator improve the biocompatibility of PMMA. The improvement was significant enough to induce unique peri-cement osteogenic behavior beyond simple increases in bone morphometric parameters such as bone volume. To the best of our knowledge, this is the first report of bone formation occurring at the resinous material interface, or *contact osteogenesis*. As described in the product manuals, PMMA-BPO is usually considered a space filler rather than an adhesive or a mechanical retention instead of an integration. The observed contact osteogenesis together with the supporting in vitro results indicate that the newly tested 4-META/MMA-TBB is osteoconductive, implying that this material could be an effective osseointegrating bone cement.

**Figure 13:**
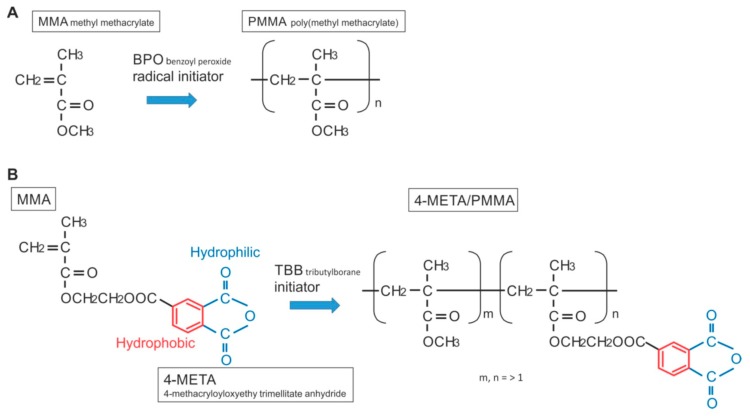
Structure and chemical formula during polymerization of PMMA-BPO and 4-META/MMA-TBB materials used in this study.

The chemical reaction during polymerization for PMMA-BPO and 4-META/MMA-TBB is shown in Figure 13. The cytotoxicity of PMMA-BPO resin was greater than expected; as many as 42% of osteoblasts did not survive 24 h after seeding on the material. This was most likely due to the significant and long-lasting production of polymerization radicals as theoretically anticipated from the chemical reaction and as demonstrated by ESR. By contrast, 80% of osteoblasts survived on 4-META/MMA-TBB resin, consistent with the ESR results showing significantly less polymerization radical production, ranging from 1/25th that of PMMA-BPO at the early stages to 1/6th that of PMMA-BPO at the later stages of polymerization. These results and interpretation were further supported by the result of NAC-incorporated PMMA-BPO showing significant improvement of its biocompatibility. NAC is known to scavenge free radicals within resin materials during polymerization. Of great note, NAC did not show a significant impact on improving 4-META/MMA-TBB, indicating that there were not much free radicals to scavenge in the material.

**Figure 14:**
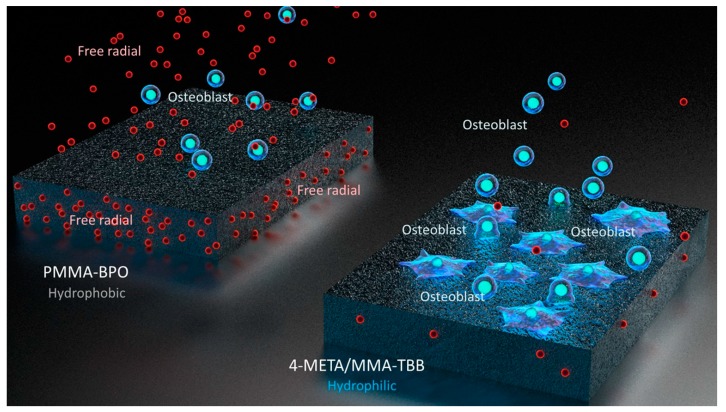
Schematic diagram of unique physicochemical property of 4-META/MMA-TBB characterized by a hydrophilic surface and minimal free radical production during polymerization, which enhances osteoblastic attachment and subsequent osteogenic function.

Interestingly, the difference in the number of attached cells between PMMA-BPO and 4-META/MMA-TBB 24 h after seeding was even larger than the difference in the percentage of viable cells at the same timepoint. This indicates that viable cells did not necessarily attach to PMMA-BPO resin, or, in other words, 4-META/MMA-TBB facilitated cell attachment, which can be considered another important benefit of 4-META/MMA-TBB. One possible explanation for this enhanced cell attachment was that adding the 4-META unit provided the material with hydrophilic ends as shown in the chemical formula (Figure 13). The hydrophilicity/hydrophobicity tested in this study not only demonstrated exclusive hydrophilicity of 4-META/MMA-TBB immediately after mixing as predicted from the chemical formula, but also sustained hydrophilicity under wet conditions. This condition-dependent phenomenon was exclusive to 4-META/MMA-TBB. The PMMA-BPO resin was consistently hydrophobic from immediately after mixing to up to 24 h after mixing, regardless of being dry or wet. Considering that bone cement is usually used under wet conditions such as in the bone marrow cavity (as in the present in vivo experiment), 4-META/MMA-TBB may maintain its hydrophilicity and therefore bioactivity during the critical time of initial healing. In summary, 4-META/MMA-TBB is characterized by newly rendered hydrophilicity and inherently suppressed free radical production and illustrated in Figure 14.

The biological effects of hydrophilicity have been reported for various biomaterials. Although the precise mechanism is unknown and the effect may be material- and cell type-dependent, hydrophilicity of material surfaces seems to have positive effects on initial cellular behaviors [26,39,40,41,42]. UV light-induced hydrophilic titanium promotes the recruitment, attachment, and spread of osteoblasts [26,29,30]. Similarly, hydrophilic zirconia and Co-Cr alloy enhances the initial behavior and response of osteoblasts compared to hydrophobic zirconia and Co-Cr alloy, respectively [41,43,44,45,46]. Hydrophilic titanium also effectively attracts and retains proteins and other cell types such as endothelial cells [47,48]. There are few reports of the effect of hydrophilicity/hydrophobicity of resinous materials. Here we revealed the positive effect of a hydrophilic monomer in a PMMA-based material on the initial behavior of osteoblasts and subsequent osteogenesis.

The in vivo anchorage of an implant in bone is the most pertinent variable with respect to the clinical capacity of bone cement as an implant-immobilizing material. The push-in test showed that the strength of bone integration for 4-META/MMA-TBB was 4-times greater than PMMA-BPO at week 2 during the early stage of healing and 7-times greater at later-stage week 4. This biomechanical benefit was first corroborated by SEM morphological and EDX elemental analyses, which showed robust bone formation around 4-META/MMA-TBB resin. The dual bone morphometric analyses of microCT and histology further supported the biomechanical findings by demonstrating a significant increase in bone volume and percentage of bone-cement contact around 4-META/MMA-TBB both for cortical bone and the bone marrow cavity. Three major interpretations of the bone morphometric analyses are worth highlighting. First, the advantage of 4-META/MMA-TBB over PMMA-BPO was generally more pronounced at week 2 than week 4, indicating that 4-META/MMA-TBB not only elevated the quantity of de novo osteogenesis but also expedited the process. In fact, bone volume and percentage of bone-cement contact for 4-META/MMA-TBB at week 2 already outperformed PMMA-BPO at week 4. Secondly, robust osteogenesis around 4-META/MMA-TBB resin compared to PMMA-BPO seemed to be more pronounced in the bone marrow cavity than cortical bone, indicating an even greater advantage of 4-META/MMA-TBB resin to promote de novo bone formation than simply a process of remodeling adjacent to innate bone. Thirdly, the increased percentage of bone-cement contact for 4-META/MMA-TBB was attributable not only to the increased bone volume but also to substantially reduced soft tissue interposition between the de novo bone and cement, indicating that the inflammatory reaction leading to granulation tissue formation was significantly diminished around 4-META/MMA-TBB, which we believe was primarily due to substantially less free radical production.

Considerable efforts have been made to develop bioactive bone cements, since a bioactive material would provide tremendous clinical benefits with regards to the longevity and success of metallic implants [49,50]. One approach has been to add a bioactive agent. Peri-cement bone formation and/or in vitro osteoblastic responses have been improved with bioactive glass [51,52,53,54,55,56], hydroxyapatite [57], or other calcium-containing molecules [58,59,60,61,62,63,64]. These studies adopted the strategy of literally adding bioactivity to bone cement materials via additives, but this approach does not fundamentally improve material biocompatibility. In fact, a potential reduction in cyto-compatibility or detailed mechanisms for an increased bone reaction were rarely assessed in these studies. As conducted in the present study, there was an effective attempt to increase biocompatibility of PMMA-based bone cement by scavenging polymerization radicals using anti-oxidants such as NAC. The addition of N-acetyl cysteine, an anti-oxidant amino acid, to PMMA-BPO bone cement significantly reduced the amount of active radical produced during polymerization and allowed peri-cement bone formation [23]. However, the present study is the first attempt to reduce the cytotoxicity of PMMA-based materials by implementing a different polymerization mechanism.

The development of improved bone cements is a pressing issue because of the increasing use and applications of bone cement in our aging society. Despite the above-mentioned efforts, bone cement materials, i.e., PMMA-based materials, have not seen significant improvements [50]. Furthermore, there is another biological imperative to improve cements. In humans, antioxidant and detoxification capacities diminish with age. Circulating glutathione, a major oxidant scavenging mechanism in humans, is 17% lower in people aged 40–59 years and 45% lower in those aged 60–79 years than those aged 20–39 years [65]. There are unique biological demands of an aging society that necessitate more biocompatible bone cements.

In addition to a lack of osteoconductivity, currently used bone cements have severe adverse systemic effects such as cardiovascular compromise and immediate mortality [18,19,66]. Current PMMA-based bone cements are considered bio-tolerant. Our results for PMMA-BPO cement confirm this. However, 4-META/MMA-TBB cement showed remarkably reduced polymerization radical production, leading to greater survival of osteoblasts, a beneficial functional phenotype, and eventually successful peri-cement bone formation with minimum soft tissue intervention, suggesting the advent of a biocompatible and osteoconductive resin. These highly promising results warrant immediate pre-clinical and clinical validation.

## 4. Materials and Methods 

### 4.1. Polymer Preparation and Characterization

PMMA-BPO resin was prepared by mixing the recommended ratio of powder and liquid (0.53 g powder and 0.25 g liquid; powder:liquid ratio (wt) = 40:18.88; Endurance MV, DePuy Orthopaedics, Warsaw, Indiana) for 30 s, placed flat and thin as even as possible in a well of a 12-well cell culture plate (22 mm in diameter), and left until it became doughy before cell culture. For 4-META/MMA-TBB resin, 4-META was added to MMA in a wt/wt ratio of 5% to make a monomer mix. Then, 0.6 g PMMA powder, monomer mix, and TBB initiator were mixed in a wt % ratio of 47:49:4 for 30 s and placed in a well. 4-META, MMA, PMMA, and TBB were manufactured and provided by Sun Medical (Moriyama, Japan). For in vivo implantation, resin implants in cylindrical form (2 mm length, 1 mm diameter) were made from PMMA-BPO and 4-META/MMA-TBB before surgery.

To determine the role of free radical production on biocompatibility of the resin materials, we prepared the experimental N-acetyl cysteine (NAC)-added materials. The free radical-scavenging function of NAC has been reported extensively [23,67,68,69,70]. NAC was prepared as 1M stock solution in HEPES buffer whose pH was adjusted to 7.2. The NAC solution was mixed with MMA liquid before mixing with PMMA powder to make a final concentration of 5 mM.

Polymerization behavior of the two materials was evaluated in multiple ways. First, the degree of polymerization was evaluated using Fourier transform infrared spectroscopy (FT-IR) (Spectrum100; PerkinElmer). The degree of polymerization was evaluated by the increasing number of repeat units of the polymer chain during polymerization. Specifically, the ratio of 1720 cm^−1^ (C=O) absorbance and 1640 cm^−1^ (C=C) was measured 1 and 24 h after mixing. The number of C=O is known unchanged during polymerization, while the number of C=C reduces. Therefore, the degree of polymerization was calculated as the reduction rate of 1640 cm^−1^ (C=C) absorbance. The temperature of the materials during polymerization was measured by using differential scanning calorimetry (DSC-60, Shimazu, Kyoto, Japan). The surface hardness of the materials was measured by Vickers testing (HM-103, Mitsutoyo, Kawasaki, Japan) at 1 and 24 h after mixing. Lastly, surface morphology of the polymerized materials was examined by scanning electron microscopy (SEM; Nova 230 Nano SEM, FEI, Hillsboro, OR, USA).

### 4.2. Osteoblastic Cell Culture

Bone marrow cells isolated from the femurs of 8-week-old male Sprague-Dawley rats were placed in alpha-modified Eagle’s medium supplemented with 15% fetal bovine serum, 50 µg/mL ascorbic acid, 10^−8^ M dexamethasone, 10 mM Na-*ß*-glycerophosphate and antibiotic-antimycotic solution containing 10,000 units/mL penicillin G sodium, 10,000 mg/mL streptomycin sulfate and 25 mg/mL amphotericin B. Cells were incubated in a humidified atmosphere of 95% air, and 5% CO_2_ at 37 °C. At 80% confluency, the cells were detached using 0.25% trypsin-1 mM EDTA-4Na and seeded onto either PMMA-BPO resin or 4-META/MMA-TBB resin at a density of 3 × 10^4^ cells/cm^2^. The culture medium was renewed every 3 days. 

### 4.3. Cell Viability Assay

A flowcytometry-based cell viability test was performed using the cells after a 24-h incubation. The test was similar to the method used in our previous studies [67,71] and based on the flow cytometric detection of annexin V binding and propidium iodide (PI) staining (Annexin V-FITC Kit, BD Bioscience, San Jose, CA, USA). Annexin V is known to bind to phosphatidylserine (PS) and PI to DNA when the cell membrane was dismantled. The intensity of PI staining (y-axis) was plotted against annexin-FITC intensity (x-axis). Viable cells were observed in the lower left quadrant (annexin V negative/PI negative), apoptotic cells in the lower right quadrant (annexin V positive/PI negative), necrotic cells in the upper left quadrant (annexin V negative/PI positive), and late necrotic cells in the upper right quadrant (annexin V positive/PI positive).

### 4.4. Morphology and Spreading Behaviors of Osteoblasts

Spreading behavior and cytoskeletal arrangement of osteoblasts seeded onto resin materials were examined using confocal laser scanning microscopy. At 24 h after seeding, cells were fixed in 10% formalin and stained using fluorescent dye rhodamine phalloidin (actin filament, red color; Molecular Probes, Eugene, OR, USA). To observe the intracellular expression and localization of vinculin, a focal adhesion protein, cells were additionally stained with mouse anti-vinculin monoclonal antibody (Abcam, Cambridge, MA, USA), followed by FITC-conjugated anti-mouse secondary antibody (Abcam). The area, perimeter, and Feret’s diameter, and the density of rhodamine- and vinculin-positive areas were quantified using an image analyzer (ImageJ, NIH, Bethesda, MD, USA).

### 4.5. Cell Attachment and Proliferation Assays

The initial attachment of cells to resin materials was evaluated by measuring the number of cells attached to the surfaces after 3 and 24 h of incubation. The density of propagated cells was measured on day 2 of culture. These measurements were performed using a tetrazolium salt (WST-1)-based colorimetric assay (WST-1; Roche Applied Science, Mannheim, Germany). Each culture well was incubated at 37 °C for 4 h with 100 μL WST-1 reagent. The amount of formazan produced was measured at 420 nm using an enzyme-linked immunosorbent assay (ELISA) reader (Synergy HT, BioTek Instruments, Winooski, VT, USA). The proliferative activity of cells was also measured by incorporating BrdU during DNA synthesis. On day 2 of culture, 100 μL of 100 mM BrdU solution (Roche Applied Science, Penzberg, Germany) was added to the culture wells followed by incubation for a further 10 h. After trypsinizing the cells and denaturing DNA, cultures were incubated with an anti-BrdU conjugated with peroxidase for 90 min and reacted with tetramethylbenzidine for color development. Absorbance was measured at 370 nm using an ELISA reader.

### 4.6. Alkaline Phosphatase (ALP) Activity

ALP activity was examined on day 7 using image-based assays. Cultured cells were washed twice with Hanks’ solution and then incubated with 120 mM Tris buffer (pH 8.4) containing 0.9 mM naphthol AS-MX phosphate and 1.8 mM fast red TR for 30 min at 37 °C. The ALP-positive area on the stained images was calculated as (stained area/total dish area) × 100 (%) using an image analyzer (ImageJ).

### 4.7. Mineralizing Capability

Von Kossa stain was utilized to determine the mineralizing capability of osteoblasts. The culture on day 14 were fixed using 50% ethanol/18% formaldehyde solution for 30 min. The cultures were then incubated with 5% silver nitrate under UV light for 30 min. Finally, the cultures were washed twice with ddH_2_O and incubated with 5% sodium thiosulfate solution for 2–5 min. The mineralized nodule area defined as (stained area/total dish area) × 100 (%) was measured using a digitized image analysis system (ImageJ).

### 4.8. Real-Time Quantitative Polymerase Chain Reaction (qPCR)

Gene expression was analyzed by qPCR on days 3 and 10. Total RNA was extracted from cells using TRIzol (Invitrogen, Carlsbad, CA, USA) and Direct-zol RNA MiniPrep kit (Zymo Research, Irvine, CA, USA). Extracted RNA was reverse-transcribed into first-strand cDNA using SuperScript III Reverse Transcriptase (Invitrogen). The quantitative PCR reaction was performed in a 20 μL volume containing 90 ng cDNA, 10 μL TaqMan Universal Master Mix II, and 1 μL TaqMan Gene Expression Assay using the QuantStudio 3 Real-Time PCR System (Thermo Fisher Scientific, Canoga Park, CA, USA) to quantify expression of type I collagen, osteopontin, and osteocalcin, mRNA. GAPDH expression was used as the endogenous control.

### 4.9. Animal Surgery

Ten-week-old male Sprague-Dawley rats were anesthetized by inhalation of 1%–2% isoflurane. The legs were shaved and scrubbed with 10% povidone-iodine solution and the distal aspects of the femurs were carefully exposed by skin incision and muscle dissection. The flat surfaces of the distal femurs were selected for implant placement. An implant site was prepared 9 mm from the distal edge of the femur by drilling with a 0.8 mm round burr followed by reaming to expand the diameter of the hole to 1.7 mm. Profuse irrigation with sterile isotonic saline solution was used for cooling and cleaning. One PMMA-BPO cylinder (2 mm in length and 1 mm in diameter) was placed in the right femur after coating with a freshly prepared 20 μg of PMMA-BPO resin, while one 4-META/MMA-TBB cylinder was placed in the left femur after coating with 20 μg of freshly prepared 4-META/MMA-TBB resin. Surgical sites were then closed in layers. Muscle and skin were sutured separately with resorbable suture thread. All of the experiments were performed following the protocol approved by The Chancellor’s Animal Research Committee at the University of California at Los Angeles and followed the PHS Policy for the Humane Care and Use of Laboratory Animals and the UCLA Animal Care and Use Training Manual guidelines (ARC #2005-175-41E, approved on 30 January 2018).

### 4.10. In Vivo Biomechanical Implant Push-In Test

The method used to assess the biomechanical strength of bone-implant integration is described elsewhere [23,72]. After 2 and 4 weeks of healing, femurs containing a cylindrical implant were harvested and embedded into auto-polymerizing resin with the top surface of the implant level. The testing machine (Instron 5544 electro-mechanical testing system, Instron, Canton, MA, USA) equipped with a 2000 N load cell and a pushing rod (diameter = 0.8 mm) was used to load the implant vertically downward into the bone marrow cavity at a crosshead speed of 1 mm/min. The push-in value was determined by measuring the peak of the load-displacement curve.

### 4.11. Morphological and Elemental Analyses of Implant/Tissue Complex

After the push-in test, implants were carefully exposed and soaked in agitated water for one hour and dried under heat and vacuum. After being carbon sputter-coated, the specimens were examined by scanning electron microscopy (SEM). The elemental composition of the implant/tissue complex was analyzed by energy dispersive X-ray spectroscopy (EDX) (UltraDry EDS Detector and Noran System 6, Thermo Fisher Scientific).

### 4.12. MicroCT Assessment for Bone Morphometry

We used three samples for the cell culture studies, except for the surface analysis which was evaluated in five samples; the method used for the assessment of bone formation around implants and resin materials has been validated and described elsewhere [23,28,73]. The femur-implant specimens, fixed in 10% buffered formalin, were scanned in a desktop μCT machine (μCT 40, SancroMedica, Bassersdorf, Switzerland) with an isotropic resolution of 8 μm. Six hundred CT slices were captured along the long axis of the implant at an X-ray energy level of 70 kVp with a current of 114 μA. Grayscale images were processed using a Gaussian low-pass noise filter and threshold algorithms to distinguish resin materials, mineralized bone and background. The specific thresholds for resin materials and bone tissue were determined by imaging the original structures.

In this experiment, the superior halves of the implants were considered as the cortical bone zone, while the apical halves the bone marrow zone. Therefore, bone morphometry was separately conducted in top 0.5 mm (cortical zone) and bottom 0.5 mm (bone marrow zone). The area of interest was set as a circumferential area within 200 μm from the resin interface based on a previous study [74]. Bone volume/tissue volume (BV/TV), trabecular thickness (Tb.Th), and trabecular number (Tb.N) were analyzed.

### 4.13. Histological Preparation

The femurs containing implants were harvested at weeks 2 and 4 of healing and fixed in 10% buffered formalin at 4 °C for 2 weeks. Specimens were dehydrated in an ascending series of alcohol rinses and embedded in light-curing epoxy resin (Technovit 7200VLC, Heraeus Kulzer, Wehrheim, Germany) without decalcification. Embedded specimens were sawed perpendicular to the longitudinal axis of the cylindrical implants at a site between 0–0.5 mm from the apical end of the implant (bone marrow zone) and a site between a site 0–0.5 mm from the top end of the implant (cortical zone). Specimens were ground to a thickness of 30 µm with a grinding system (Exakt Apparatebau, Norderstedt, Germany). Sections were stained with Goldner’s trichrome stain and observed by light microscopy.

### 4.14. Histomorphometry

Histological images were subjected to computer-based histomorphometric measurements (ImageJ). To identify the tissue structure details, microscopic magnification up to 200× was used. Based on the previous establishment and validation [26,74], the following variables were analyzed at each of the cortical and bone marrow zones: Bone area (%) = (bone area in the area of interest)/(total area of the area of interest) × 100, where the area of interest was defined as the circumferential zone within 200 µm of cement surface. Bone–cement contact (%) = (sum of the length of bone–cement contact)/(circumference of the cement) × 100, where the bone–cement contact was defined as the interface where the bone tissue was located within 20 µm of the implant surface without fibrous tissue intervention.

### 4.15. Hydrophilicity / Hydrophobicity Test of Resin Surfaces

Hydrophobic/hydrophilic property of resin surfaces was examined by measuring the contact angle of 10 µl of ddH_2_O placed on the materials. The measurement was performed immediately after mixing, 15, 30, 60 min, and 24 h post-mixing under dry and wet conditions. To create wet condition, the resin surface was covered with ddH_2_O until the measurement was performed.

### 4.16. Electron Spin Resonance Spectroscopy (ESR) for Polymerization Radicals

Production of free radicals during polymerization was assessed by ESR, which has been validated and developed for the various in vitro biomedical applications [75,76,77]. Cement specimens were examined using a JES-RE 3X, X-band spectrometer (JEOL,Tokyo, Japan) connected to a WIN-RAD ESR Analyzer (Radical Research, Tokyo, Japan) at the following settings: modulation amplitude, 0.063 mT; sweep width, 5 mT; sweep time, 1 min; time constant, 0.03 s; microwave power, 8 mW; and magnetic field, 335.5 mT. The component signals in the spectra were identified and quantified as reported previously [75]. The measurement was continued up to 10 days after mixing.

### 4.17. Statistical Analysis

The number of samples was 3 for the cell culture studies including hydrophilicity/hydrophobicity test, Vickers test, and ESR, except for cytomorphometry (n=6). For in vivo experiments, the number of samples was 5 for the biomechanical push-in test, and 4 for microCT and histomorphometry. The t-test was used to examine differences between PMMA-BPO and 4-META/MMA-TBB materials; *p* < 0.05 was considered statistically significant.

## 5. Conclusions

This study determined whether the addition of 4-META into MMA and use of TBB as a polymerization initiator instead of BPO significantly improve PMMA-based materials. A greater number of osteoblasts survived on 4-META/MMA-TBB than on PMMA-BPO. The significantly suppressed osteoblastic functional phenotypes on PMMA-BPO materials were significantly restored on 4-META/MMA-TBB. De novo bone formation occurred around 4-META/MMA-TBB with minimal soft tissue interposition, while bone formation was fragmentary and discrete around PMMA-BPO. Nearly the entire surface of PMMA-BPO was covered with soft tissue, and there was no direct contact of bone and PMMA-BPO. The implant anchorage of 4-META/MMA-TBB was 4-times stronger than that of PMMA-BPO at the early stage of healing and 5-times stronger at later stages. The surface of 4-META/MMA-TBB showed long-lasting hydrophilicity, while the PMMA-BPO was consistently hydrophobic. Free radical production during polymerization of 4-META/MMA-TBB was 1/25th to 1/10th that of PMMA-BPO during the first 24 h. These results suggest that 4-META/MMA-TBB is osteoconductive and deserves further study with a view for therapeutic use.

## Abbreviations

BCIS	Bone cement implantation syndrome
BPO	Benzoyl peroxide
ESR	Electron spin resonance
MMA	Methyl methacrylate
NAC	N-acetyl cysteine
PMMA	Polymethyl methacrylate
TBB	Tri-n-butyl borane
4-META	4-methacryloyloxylethyl trimellitate anhydride
